# Limitations and Innovative Application Methods of Surfactants for Solubilization of Poorly Water-Soluble Drugs

**DOI:** 10.2174/0115672018299592240524074005

**Published:** 2024-05-29

**Authors:** Gang Jin, Jie Wang, Jie Xu, Qing Jin, Jian-Fei Xue, Lin-Han Li

**Affiliations:** 1 Department of Pharmacy, School of Chemical and Pharmaceutical Engineering, Jilin Institute of Chemical Technology, Jilin 132022 , PR China;; 2 Party and Government Office, Jilin Institute of Chemical Technology, Jilin 132022 , PR China;; 3 Department of Materials Science and Engineering, Korea University, Seoul 02841 , Republic of Korea

**Keywords:** Limitations, surfactants, poorly water-soluble drug, innovative application methods, solubilization, micelle

## Abstract

Poor solubility of drugs leads to poor bioavailability and therapeutic efficiency. A large proportion of drugs that are not developed and marketed for use by patients are due to their extremely low solubility. Therefore, improving the solubility of poorly water-soluble drugs is one of the most important aspects of the field of drug research. With the continuous development of more and more formulation techniques and excipient applications, the solubility of poorly water-soluble drugs can be improved to a certain extent to obtain better pharmacokinetics and pharmacodynamics, including pH microenvironment regulation technology, inclusion complex, solid dispersion, nanotechnology, and application of surfactants. However, the most widely used among them is the application of surfactants. This technique can reduce the surface tension, improve wettability, and have a remarkable solubilizing ability after forming micelles. However, surfactants have also been found to possess certain limitations in solubilization. In this review, the factors affecting the solubilization of surfactants and limiting their application have been summarized from several aspects. These factors include drugs, additives, and media. Some ideas to solve these application limitations have also been put forward, which can lay a foundation for the wider application of surfactants in the future.

## 1 INTRODUCTION

During the development of new drugs, the problem of low bioavailability of poorly water-soluble drugs is one of the most important factors limiting the application of the drug to patients. Currently, about 40% of orally administered drugs are categorized as practically insoluble in water [1]. For drugs with poor solubility, the bioavailability after oral administration can be improved by increasing their solubility and dissolution properties [2-5].

Several formulation technologies have been used to increase the dissolution rate of poorly water-soluble drugs, including solid dispersion [6-9], inclusion complex [10-13], micronization techniques [14-17], salt formation [18-20], solubilization in lipid-based preconcentrates or colloidal lipid carrier systems [21, 22], and application of surfactants [23-29]. Among these, the application of surfactants to solubilize poorly water-soluble drugs through micellization and to improve the wetting of hydrophobic drugs during dissolution has been most widely adopted. Moreover, surfactants are used in many ways and can be used in combination with other solubilization techniques in addition to being used alone [30-40].

Surfactants form aggregates in aqueous as well as in non-aqueous solutions beyond a certain concentration called micelles, and this required concentration for this phenomenon is known as Critical Micelle Concentration (CMC), which has found various applications in different fields [41-45]. As depicted in Fig. (**1**), surfactants are organic compounds that are amphiphilic and composed of hydrophilic heads and hydrophobic tails. When the concentration of surfactant molecules in a system is low, they adsorb onto surfaces or interfaces. At concentrations above CMC, they can significantly increase the water solubility of low-soluble drugs due to the formation of micelles; the aqueous solubility increases linearly with the surfactant concentration [46, 47].

Surfactants can be classified into four categories according to their dissociation in water. Anionic surfactants dissociate in water to generate hydrophilic anions. For example, sodium lauryl sulfate surrounded by water molecules is dissociated into two parts, ROSO_2_-O ^-^ and Na ^+^. The negatively charged ROSO_2_-O ^-^ has surface activity. The cationic surfactant is a kind of surfactant with a positive charge, which connects with the lipophilic group. The lipophilic group is generally a long carbon chain hydrocarbon group. Most of the hydrophilic groups are cations with nitrogen atoms, and a few are cations with sulfur or phosphorus atoms. Anions in molecules do not have a surface activity and are usually single atoms or groups, such as chlorine, bromine, acetate ions, *etc*. The cationic surfactant has a positive charge, which is opposite to the charge of the anionic surfactant. When they are used together, the precipitation takes place and the surface activity is lost. Nonionic surfactants do not dissociate in aqueous solution. These are less irritant than anionic and cationic surfactants. Amphoteric surfactants are mild in nature and they can be anionic, cationic, or nonionic, depending on the pH of the water [48]. The properties of commonly used surfactants are presented in Table **1** [49-58].

As shown in Fig. (**2**), the use of a small amount of surfactant can change the sink conditions and improve the wettability by surface adsorption. When the concentration is higher than the CMC, the drug is encapsulated in the core of micelles, which has a more significant solubilization effect. However, it still has certain limitations in application, such as safety problems caused by excessive use, the influence of different excipients and drugs on CMC, and the stability of surfactants in different pH environments. Therefore, this review has outlined the factors affecting the solubilization of surfactants and some ideas to solve the negative phenomena of solubilization.

## 2 EFFECT OF DRUG TYPES ON SOLUBILIZATION OF SURFACTANTS

Surfactants do not improve the solubility of all poorly water-soluble drugs due to differences in drug type and chemical structure. For example, for cationic drugs, the addition of an anionic surfactant to the formulation does not always obtain improved solubility results. Even during the preparation and dissolution process of nanosuspension, insoluble precipitates are formed due to the intermolecular electrostatic interactions between partially ionizable anionic drugs and oppositely charged surfactants, which lead to the low drug content, increased particle size, the formation of rough and irregular crystals, and decreased dissolution [59]. Meanwhile, since the concentration of the surfactant in the medium at that time is much lower than the CMC, the interaction between the drug and the surfactant under certain conditions leads to the formation of an insoluble complex, which ultimately results in a reduced dissolution rate of the drug. For instance, anionic surfactant Sodium Lauryl Sulfate (SLS) was used in ritonavir tablet formulation. It was revealed that a poorly soluble salt [RTV^2+^][LS^−^]_2_ was formed in an acidic environment when a small amount of SLS was added. Subsequently, the phenomenon of dissolution rate reduction was observed [60]. In addition, the dissolution rate of cationic drugs is also affected by the insufficient amount of SLS in the dissolution medium. Desolubilization of cationic drug substance was observed at pH 4.5 in the presence of SLS at concentrations below CMC, which was attributed to the formation of an insoluble di-dodecyl sulfate salt between SLS and the drug [61]. However, not all electrostatic interactions between oppositely charged drugs and surfactants reduce the solubility of the drugs. A study investigated the effects of three types of surfactants, anionic SLS, cationic Dodecyltrimethylammonium Bromide (DTAB), and nonionic n-dodecyl octa(ethylene oxide) (C_12_EO_8_) on the solubilization of the anionic drug Ibuprofen (IBU). The results showed that the anionic surfactant SLS exhibited the worst IBU solubilization performance due to the electrostatic repulsion between the drug and the surfactant head group, while the cationic surfactant Dodecyltrimethylammonium Bromide (DTAB) provided the highest IBU molar solubilization ability due to electrostatic attraction. For the non-ionic C_12_EO_8_ micellar solution, a solubility curve similar to DTAB was observed. The increased solubility of the drug in the C_12_EO_8_ micellar solution was found to be the result of the interaction between IBU and the head group of PEO surfactant and also due to the lower CMC [51]. Some studies have also shown the dissolution rate to be slowed down by the surfactant [62-64].

In addition, amphiphilic drugs are also self-associating and behave like surfactants. Therefore, they have a significant impact on the micellization ability of surfactants. For example, the amphiphilicity of Diphenhydramine Hydrochloride (DPH) is due to the presence of diphenylmethane groups. As shown in Table **2**, when DPH was mixed with CTAB or Sodium Dodecylbenzene Sulfonate (SDBS), respectively, under different stoichiometric mole fractions, the CMC of the mixed system changed significantly [65].

Therefore, relative to nonionic drugs, the investigation of the solubilization effect of surfactants on ionic drugs no longer requires the consideration of micelle formation alone, but rather a comprehensive examination of the interconnections between the environment, drugs, and surfactants. For example, when the concentration of surfactant in the environment is lower than the CMC under specific pH conditions, it needs to be determined whether the solubilizing ability of surfactant will be affected by the formation of insoluble complexes between partially dissociated drug and surfactant due to the effect of electrostatic attraction between molecules with opposite charges, whether the solubilizing capacity of the surfactant will be reduced due to the repulsive interaction between the drug ions and the surfactant head between the drug and the surfactant of the same charge under specific pH conditions, whether there will be an interaction between ionic drug and non-ionic surfactant to drive the drug to the outside of the micellar core to reduce the surfactant CMC and thus increase the solubilising capacity of the surfactant at a given pH, and whether amphiphilic drugs and surfactants will be able to reduce the CMC due to the formation of mixed micelles at specific pH conditions, thereby increasing the solubilising capacity of surfactants.

## 3 EFFECT OF ADDITIVES ON CMC OF SURFACTANTS

The CMC is an important feature of surfactants. It is a measure of the activity of surfactants. The smaller the CMC, the lower the concentration of surfactant required to form micelles. Only at a concentration above the CMC can the surfactant’s effect be fully realized. However, there are many factors that affect the CMC, for example, pH, temperature, and other additives. In particular, the addition of additives has the most significant effect. There have been many studies on the effect of various additives on the solubilizing ability of surfactants [66-75]. Since in most cases, it is difficult to prepare a dosage form that meets quality standards by merely adding surfactant alone, it is often necessary to add other additives, such as inorganic salts and organic polymers, to the formulation. Table **3** shows the effect of different concentrations of salt on the CMC value of Sodium Dodecyl-benzenesulfonate (SDBS). The addition of salt significantly changed the CMC value of the surfactant [76]. For different types of surfactants, the addition of the above two substances may tend to have different effects. The CMC of the surfactant can be significantly increased in a specific environment. It may lead to difficult micelles formation and low solubilization.

If the ionic surfactants are selected, adding an appropriate amount of an inorganic salt to the formulation may help to reduce the CMC of the surfactant, thereby further increasing the solubility of the drug [77-79]. For example, in the anionic surfactant Sodium Lauryl Sulfate (SLS), the mechanism may be mainly due to the head of SLS, which is positively charged. The similarly charged particles may often produce repulsive force when aggregated, making it difficult to aggregate more SLS to form micelles. Adding inorganic salts may help to reduce the repulsive force and increase the possibility of SLS aggregation, making it easier to form micelles. Moreover, the addition of inorganic salts may not play any role with nonionic surfactants, and also the stability may be affected by the addition of unnecessary excipients.

The effects of different polar organic compounds on the CMC of surfactants are significantly different. For example, in the case of SLS, SLS is adsorbed on the chain of the polar polymer, and the repulsion force with free SLS leads to hindrance in further adsorption. If the chain of the polymer is long enough, the repulsive force between them will be significantly reduced. It will thus be easier for SLS to aggregate to form micelles for solubilization. If strong polar organic compounds with low molecular weight are introduced, they can destroy water structure and make micelles difficult to form and increase CMC [77, 78]. The effects of various additives on the formation of surfactant micelles are shown in Fig. (**3**).

In addition to the above methods, the more common application is the mixed surfactant system, which reduces the CMC value through the electrostatic or hydrophobic interaction of different types of surfactants so that the drug can be more easily encapsulated in the micelles to achieve a good solubilization effect. A study showed that in a mixed system of Brij 58 and ibuprofen, an increase in the molar fraction of Brij 58 reduced the CMC of the system, and with the introduction of NaCl, the CMC of the system could be further reduced mainly due to the introduction of salt because of the reduction in the surface charge of the micelles [44]. Another study has demonstrated the mixture of surfactants sodium bis(2-ethylhexyl) sulfosuccinate (Na-AOT) and Benzyl-n-hexadecyldimethylammonium Chloride (BHDC) to form a new type of catanionic surfactant bis(2-ethylhexyl) sulfosuccinate-benzyl-n-hexadecyldimethylammonium (AOT-BHD) by removing their counter ions. The AOT-BHD showed low polarity, high viscosity, and higher electron donor capacity [80].

It has been seen that the reasonable introduction of additives can significantly improve the solubilising ability of surfactants, the mechanism of which is mainly attributed to how the additives reduce the CMC of surfactants. For example, the introduction of inorganic salts can significantly reduce the repulsive force generated by the aggregation of charged particles of ionic surfactants, which can, in turn, increase the possibility of surfactant polymerisation and ultimately reduce the CMC. It should be noted that the introduction of inorganic salts has no effect on nonionic surfactants. At the same time, there is a significant difference in the effect of the introduction of organic compounds of different polarities on the CMC under specific conditions. Ionic surfactants can be adsorbed on the chains of polar polymers and have a certain repulsive force with the unadsorbed free surfactant, which can be significantly reduced if the chains are sufficiently long to form micelles more easily and significantly increase their solubilising capacity. In contrast, the introduction of low molecular weight polar organic compounds can disrupt the water structure and make micelles difficult to form. In addition to the above additives, the introduction of drugs with specific structures and additional surfactants can also significantly reduce the CMC.

## 4 EFFECT OF DISSOLUTION MEDIA ON SOLUBILIZATION OF SURFACTANTS

When the formulation contains the cationic drug and an anionic surfactant and when the concentration of the surfactant is lower than the CMC in the dissolution medium, the dissolution rate of the drug may be significantly different in various pH media. Because the degree of protonation of drugs in various pH media is different, the drug after protonation may form an insoluble salt with surfactant [81-83]. It can also result in a decrease in the solubilization of the surfactant. Some surfactants also are not very stable in pH buffer. For example, sodium dodecyl sulfate could be hydrolyzed in an acidic medium, which led to the decomposition of alkyl sulfates into fatty alcohols and sodium sulfate in an alkaline environment. Thus, its solubilization has been found to be limited [78]. In Table **4**, a series of studies on the negative effects of surfactants are enumerated. The interaction of drugs with surfactants and the instability of surfactants in different media may lead to a decrease in dissolution and solubility [84-87].

It can be seen that the formation of insoluble complexes in different media described above is mainly the result of intermolecular electrostatic interactions between drugs and surfactants with different charges. However, the complex may not form when the concentration of surfactant is higher than the CMC, due to the significantly improved water solubility by the drug encapsulated in the micelle. In general, it is not possible to achieve the CMC through the unlimited addition of a large amount of surfactants in both the medium and the formulation. Therefore, how to introduce small doses of surfactants to achieve the best solubilization effect is the focus of current research. The effective method at present is to introduce micellarized surfactants into the preparation process, allowing dissolved drugs to adsorb onto the surface of the micellarized surfactants, effectively preventing drug aggregation and recrystallization, improving the wettability of drugs, reducing particle size, and thereby enhancing the solubilising ability of the surfactants.

## 5 EFFECT OF MIXED MICELLIZATION ON SOLUBILIZATION OF SURFACTANTS

Mixed Micelle Systems (MMS) are often chosen when the solubilization effect of using a single surfactant is not significant, for example, binary or ternary mixed micelles. The formation of mixed micelles can significantly reduce the CMC compared to a single component. It can significantly ameliorate the micelle instability caused by dilution of the micelle system, making it easier for drugs to enter their shell-core structure during the micelle formation process, thus leading to better solubilization effects. Nowadays, substantial research emphasizes the aggregation and interactions of drugs and surfactants, surfactants and polymers, different surfactants, and different polymers in binary or ternary amphiphilic systems [88-93]. In Table **5**, a series of studies on the solubilization and release of poorly water-soluble drugs by MMS are enumerated. The future research direction is to elucidate the formation mechanism, advantages, and application drawbacks of the above MMS, and to explore a more stable, better solubilizing effect, and easier preparation of MMS [94-100].

## CONCLUSION

Improving the solubility and dissolution of poorly water-soluble drugs and meeting the quality standards are difficult to achieve with a single surfactant, and thus, the assistance of other additives is usually required. However, the following aspects should be noted when using surfactants: first, understanding of the physicochemical properties of the drug. In order to avoid the interaction of the ionic drug with the ionic surfactant under certain conditions to form a poorly soluble salt, a nonionic surfactant can be selected. Secondly, when the surfactant solubilization effect is not obvious, a certain amount of additives, such as an inorganic salt and a polar organic compound, is added according to the type of the surfactant to reduce the CMC of the surfactant in order to improve the solubilization property. The use of strong and polar low molecular weight organic compounds should be avoided when it is necessary because it leads to an increase in the CMC. Thirdly, adding an alkalizer or salt into the formulation reduces the degree of protonation of the drug, as the dissolution medium selected in a specific case cannot be changed, and the pH values of various dissolution media are different. The competition effect of hydrogen ions after alkalizer or salt addition minimizes the formation of poorly soluble salts between positively charged drugs and surfactants. Finally, due to safety issues, large amounts of surfactants should be avoided as much as possible. Also, attention should be paid to how to use a small dose of surfactant to achieve a better solubilization effect and the method of introducing the surfactant into the formulation. For instance, a little surfactant must be dissolved in a small amount of aqueous solution to make it above the CMC, and pre-incorporation of micellized surfactant by anti-solvent method is an effective way [101]. All in all, there are many factors that affect the solubilization effect of surfactants. How to use a small amount of surfactants, the targeted use of drugs, and the reasonable addition of excipients to achieve a better solubilization effect and avoid negative effects as much as possible are the focus of the application of surfactants in the solubilization of poorly soluble drugs.

## Figures and Tables

**Fig. (1):**
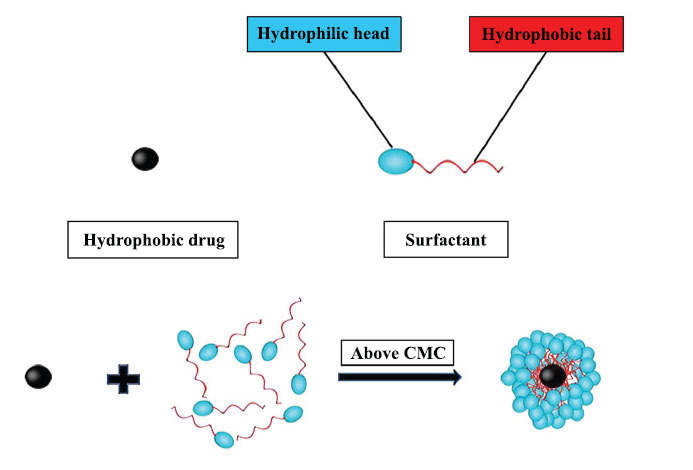
Micelle formation of the surfactants.

**Fig. (2):**
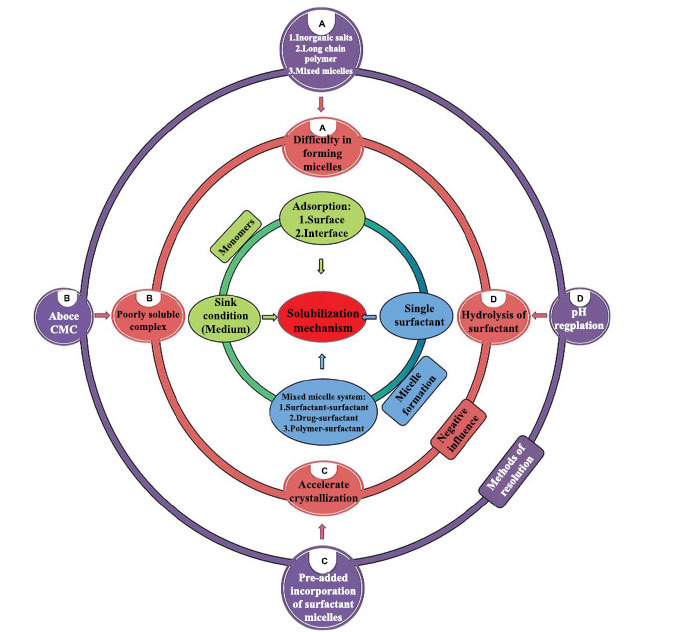
Mechanistic understanding of the solubilization effect, negative influence, and possible effects of surfactants on poorly water-soluble drugs.

**Fig. (3):**
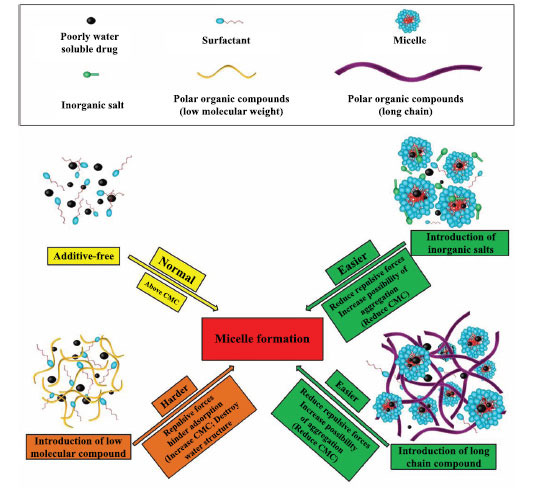
Effect of various additives on the formation of surfactant micelles.

**Table 1:** Properties of the surfactants.

**S. No.**	**Surfactant**	**Type**	**Chemical** **Structure**	**Chemical** **Formula**	**Molecular** **Weight** **(g/mol)**	**CMC**	**References**
1.	SLS	Anionic	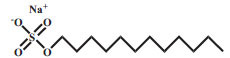	C_12_H_25_O_4_SNa	288.38	2.34 mg/mL	[49]
2.	DS	Anionic	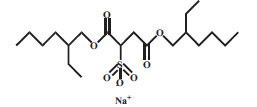	C_20_H_37_NaO_7_S	444.6	2.66 mM	[50]
3.	DTAB	Cationic	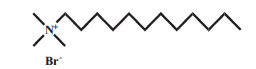	C_15_H_34_BrN	308.34	15.9mM	[51]
4.	CTAB	Cationic	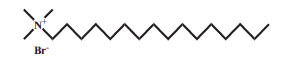	C_19_H_42_BrN	364.45	0.328 mg/mL	[52]
5.	BAC	Cationic	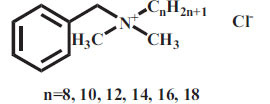	C_6_H_5_CH_2_N (CH_3_)_2_RCl (R=C_8_H_17_ to C_18_H_37_)	283.88-424.15	1.32 mg/mL	[53]
6.	Tween 80	Nonionic	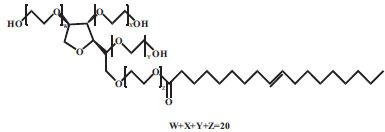	C_64_H_124_O_26_	1310	0.015 mM	[54]
7.	POX 188	Nonionic	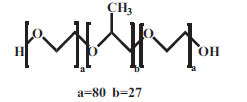	HO(C_2_H_4_O)_a_(C_3_H_6_O)_b_(C_2_H_4_O)_a_H a= 80 b= 27	8400	24–32 mg/mL 37°C; 0.48mM 25°C	[55, 56]
8.	POX 407	Nonionic	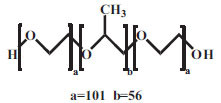	HO(C_2_H_4_O)_a_(C_3_H_6_O)_b_(C_2_H_4_O)_a_H a= 101 b= 56	12500	0.0027 mM 25°C	[56]
9.	Soluplus	Nonionic	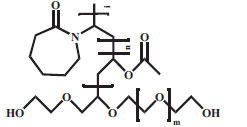	-(C_8_H_13_NO)_i_(C_4_H_6_O_2_)_n_C_5_H_10_O_3_ (CH_2_O)_m_C_2_H_4_OH	Average 118000	7.6μg/mL	[57]
10.	Vitamin E TPGS	Nonionic	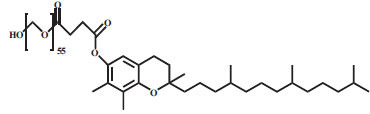	C_33_H_54_O_5_.(C_2_H_4_O)n	530.78+(44.05)n	0.02% w/w	[58]

**Abbreviations:** SLS, Sodium lauryl sulfate. DS, Sodium dioctyl sulfosuccinate. DTAB, Dodecyltrimethylammonium bromide. CTAB, Cetyltrimethylammonium bromide. BAC, Benzalkonium chloride. POX 188, Poloxamer 188. POX 407, Poloxamer 407. Soluplus, Polyethylene caprolactam-polyvinyl acetate-polyethylene glycol graft copolymer. Vitamin E TPGS, Vitamin E D-a-tocopherol polyethylene glycol 1000 succinate.

**Table 2:** CMC value of DPH + CTAB/SDBS mixed system*.

**S. No.**	**^α^DPH (DPH + CTAB)**	**CMC (mM)**	**^α^DPH (DPH + SDBS)**	**CMC (mM)**
1.	0.10	1.09	0.05	0.30
2.	0.30	1.38	0.10	0.27
3.	0.50	1.62	0.15	0.29
4.	0.70	2.46	0.90	1.97
5.	0.90	4.90	0.95	2.33

Note: *[65]. α Stoichiometric mole fraction.

**Abbreviations:** DPH, diphenhydramine hydrochloride.

**Table 3:** CMC values of sodium dodecyl-benzenesulfonate in the presence of different salt concentrations.

**S. No.**	**Salt**	**Salt Concentration**	**CMC (mM)**
1.	-	-	1.2
2.	KCl	10	0.8
3.	KCl	20	0.4
4.	KCl	100	0.15
5.	KCl	250	0.1
6.	Na_2_CO_3_	10	0.8
7.	CaCl_2_	0.1	0.8
8.	CaCl_2_	0.5	0.3

Note: *[76].

**Table 4:** Study on the solubilization of surfactants.

**S. No.**	**API**	**Surfactant**	**Reduced**	**Reason**	**Media**	**References**
1.	Trimethoprim (TMP)	SLS	Solubility	TMP-lauryl sulfate salt	0.2% NaCl, 0.01N HCL	[62]
2.	PG-300995	SLS	Solubility	Insoluble estolate salt	pH 2.0 buffer	[82]
3.	Cilostazol	SLS	Solubility	SLS decomposition	pH 6.8	[84]
4.	Ritonavir	SLS	Dissolution	Poorly soluble salt [RTV^2+^] [LS^-^]_2_	0.1N HCL, pH 1.2	[60]
5.	Propranolol hydrochloride	Arlacel 60	Dissolution	Decreased penetration rate of water into matrices	pH 1.2 and 6.8	[63]
6.	Propranolol hydrochloride	SLS	Dissolution	Formation of complex	pH 1.2 and 6.8	[63]
7.	Sulphamerazine	SLS	Dissolution	SLS hydrolysis	pH 1.2	[85]
8.	Poorly soluble drug	Docusate sodium	Dissolution	Increased viscosity due to interaction between surfactant and polymer (Povidone)	Aqueous solution containing SLS	[86]
9.	l-tetrahydropalmatine	POX 188	Bioavailability	Accelerated crystallization	pH 6.8	[87]

**Table 5:** Study on the effect of MMS on the solubilization and release of poorly water-soluble drugs.

**S. No.**	**API**	**Composition of MMS**	**Effects**	**Mechanism**	**Media**	**References**
1.	Aripiprazole	Soluplus + TPGS	Prolonged release	Strong hydrophobic interaction between aripiprazole and the inner core of the micelles	SGF/SIF	[94]
2.	Ciprofloxacin	Pluronic F108 + Pluronic L81	Increased solubility	Better encapsulation of the drug	Aqueous	[95]
3.	Hydrochlorothiazide	Sodium cholate + lecithin + Poloxamer 407/PEG 4000	Increased dissolution	Drug molecules loaded into the lipophilic core of the nanostructured micellar system	0.1N HCl, pH1.2	[96]
4.	Mefenamic acid	Stevia-G + LTAC	Increased solubility	Mixed micelle formation between stevia-G and LTAC *via* hydrogen bonding and electrostatic interaction	Aqueous	[29]
5.	Silymarin	Normal Pluronic (P84) + reverse Pluronic (10R5)	Increase solubility/ Controlled release	Decreased CMC/hydrophobic nature of P84	Aqueous/pH.4 buffer	[97]
6.	Vilazodone	Phospholipid + Brij58/Labrasol	Increased solubility/dissolution	Incorporation of the non-polar moiety of the drug in the interior core of the phospholipid mixed micelle/formation of nanoparticle size of micelles	Deionised water/0.1MHCl/0.1 M PBS	[98]
7.	Carvedilol	Pluronic® F127 + TPGS + Cys	Increased solubility/dissolution	Mixed micelle formation and nanoscale particle size of drug delivery system, providing a larger surface area	Ultra-pure water/pH1.45 solution	[99]
8.	Clozapine/oxcarbazepine	Pluronic L64 + Pluronic P84/Pluronic L64 + Pluronic F127	Increased solubility/controlled release	Decreased CMC, different core sizes providing a hydrophobic pool; solubilization of both in the core and the corona region, controlled concentration of one Pluronic	Double distilled water/pH7.4 buffer solution	[100]

**Abbreviations:** SGF, Simulated gastric fluid; SIF, Simulated intestinal fluid; Stevia-G, Transglycosylated stevia; LTAC, Lauryltrimethylammonium chloride; TPGS, D-α-tocopheryl polyethylene glycol 1000 succinate; Cys, L-cysteine hydrochloride monohydrate.

## LIST OF ABBREVIATIONS

BAC: Benzalkonium Chloride
BHDC: Benzyl-n-hexadecyldimethyl-ammonium Chloride
CMC: Critical Micelle Concentration
CTAB: Cetyltrimethylammonium Bromide
Cys: L-cysteine Hydrochloride Monohydrate
DPH: Diphenhydramine Hydrochloride
DS: Sodium Dioctyl Sulfosuccinate
DTAB: Dodecyltrimethylammonium Bromide
LTAC: Lauryltrimethylammonium Chloride
MMS: Mixed Micelle Systems
POX 188: Poloxamer 188
POX 407: Poloxamer 407
SDBS: Sodium Dodecylbenzene Sulfonate
SGF: Simulated Gastric Fluid
SIF: Simulated Intestinal Fluid
SLS: Sodium Lauryl Sulfate
Soluplus: Polyethylene Caprolactam-polyvinyl Acetate-polyethylene Glycol Graft Copolymer
Stevia-G: Transglycosylated Stevia
Vitamin E TPGS: Vitamin E D-a-tocopherol Polyethylene Glycol 1000 Succinate
